# Pernicious Anemia Presenting as a Mimicker of Thrombotic Thrombocytopenic Purpura

**DOI:** 10.7759/cureus.39080

**Published:** 2023-05-16

**Authors:** Omar Alzarkali, Jane H Lee, Kathryn Bower

**Affiliations:** 1 Internal Medicine, HCA Florida Blake Hospital, Bradenton, USA; 2 Hematology and Medical Oncology, HCA Florida Blake Hospital, Bradenton, USA

**Keywords:** pernicious anemia, clinical hematology, “pancytopenia”, acute hemolytic anemia, thrombotic thrombocytopenic purpura (ttp)-like syndrome

## Abstract

A 52-year-old woman with no significant past medical history presented to the emergency room (ER) with nonspecific systemic symptoms, including fatigue, dyspnea on exertion, easy bruising, and palpitations. She was found to have significant pancytopenia. Hemolytic anemia, thrombocytopenia, and elevated PLASMIC score (6, High risk; PLASMIC = Platelet count; combined hemoLysis variable; absence of Active cancer; absence of Stem-cell or solid-organ transplant; MCV; INR; Creatinine) score at the time of presentation led to a concern for thrombotic thrombocytopenic purpura (TTP). Therapeutic plasma exchange (TPE) was deferred pending additional investigation. Workup revealed the true diagnosis of severe B12 deficiency, which would not have benefited from TPE and instead would have placed the patient at risk for harm, making the decision to defer treatment the correct and judicious approach. This is a case where anchoring on lab results may result in reaching the incorrect diagnosis. This case reminds clinicians of the importance of creating a broad differential and ensuring thorough history-taking is done for all patients.

## Introduction

Thrombotic thrombocytopenic purpura (TTP) is a well-known type of thrombotic microangiopathy (TMA) caused by severely decreased ADAMTS13 functionality [1]. ADAMTS13, the protease that cleaves von Willebrand factor (vWF) to limit platelet activation beyond physiologic requirement, is essential for maintaining homeostatic coagulation. The absence of this protein allows for the unregulated activation of vWF leading to platelet consumption and formation of microthrombosis throughout the vasculature. This eventually leads to the shearing of red blood cells resulting in intravascular hemolytic anemia. First-line treatment for patients with TTP is early therapeutic plasma exchange (TPE) that is initiated once TTP is strongly suspected [2]. Plasma exchange replaces all missing components of the patient’s plasma including ADAMTS13. Patients can present with any of the typical TTP pentad signs including thrombocytopenia, microangiopathic hemolytic anemia (MAHA), fever, acute kidney injury, and neurologic symptoms [3]. While this pentad is well-known as the classic presentation, very few patients present clinically with all five signs making accurate and timely diagnosis challenging. The only true diagnostic criterion is the serologic confirmation of reduced ADAMTS13 activity, which unfortunately may take days to return. Due to the variability in presentations and unavailability of confirmatory diagnostic criteria at the time initiation of therapy is required, a decision must be made in regard to the initiation of plasma exchange. What makes this decision challenging is the need to urgently start therapy at the time the diagnosis is suspected due to the mortality rate of untreated TTP being as high as 90%. This must be balanced with the risks associated with administering TPE such as anaphylaxis from plasma products, systemic infections from central lines, and catheter-associated thrombosis [4,5]. To assist in making this decision, the PLASMIC score can be used to predict the likelihood of reduced ADAMTS13 activity [6]. The score uses data that is typically available early in the onset of the workup, including platelet count, hemolysis, presence of active cancer/transplant, mean corpuscular volume (MCV), international normalized ratio (INR), and serum creatinine to stratify the likelihood of TTP into low, intermediate, and high probability. The score varies in sensitivity and specificity for the score range as well as the patient’s age [7]. This variability makes this test prone to false positives and negatives emphasizing the need for a comprehensive evaluation of the patient prior to making final clinical decisions.

Pernicious anemia is an autoimmune condition that results in macrocytic anemia due to impaired vitamin B12 absorption. Autoantibodies bind intrinsic factors secreted by parietal cells in the stomach blocking the protein’s primary function in assisting the absorption of the essential cofactor [8]. Lack of vitamin B12 leads to impaired DNA synthesis and therefore impaired replacement of blood cells resulting in various cytopenias [9]. Anemia secondary to nutritional deficiencies typically presents with blood work consistent with hypoproliferation, including decreased reticulocyte index. Due to the inefficient nature of erythropoiesis, the circulating macrocytic cells and growing precursors in the bone marrow are more prone to hemolysis and apoptosis respectively. The resulting serologic derangements include elevated lactate dehydrogenase (LDH), elevated indirect bilirubin, and decreased haptoglobin, which can deceive practitioners into suspecting primary hemolytic anemia as opposed to hemolysis secondary to the megaloblastic anemia [10]. This confusion can lead to misdiagnosing and initiating inappropriate therapy, delay of care, and potential harm to the patient.

## Case presentation

A 52-year-old woman presented to the ER with fatigue, dyspnea on exertion, easy bruising, and occasional palpitations for the last month. She had no prior medical history and denied recent travel, recent illnesses, or exposure to sick contacts. She did not smoke, drink alcohol, or use any illicit drugs and stated she eats a well-balanced diet. Preliminary workup in the ED demonstrated significant pancytopenia with white blood cells (WBC) of 3.9x103/mm^3^, platelet count of 25x103/mm^3^, hemoglobin 5.8 g/dL, and RBC 1.39x106/mm^3^ with an MCV of 119 fL. The blood smear demonstrated poikilocytosis 1+, basophilic stippling 1+, anisocytosis 2+, and schistocytes 1+. The remaining lab work completed on the initial presentation is included in Table 1. Additional studies ordered, which were pending, included the iron panel, serum vitamin B12/folate, and ADAMTS13 levels. The PLASMIC (Platelet count; combined hemoLysis variable; absence of Active cancer; absence of Stem-cell or solid-organ transplant; mean corpuscular volume (MCV); INR; Creatinine) score was calculated at 6, consistent with 72% probability of TTP (sensitivity 65.2%, specificity 72%). Table 2 demonstrates the breakdown of the scoring. Two units of pRBCs were ordered and a post-transfusion repeat of CBC demonstrated marked improvement of hemoglobin to 8.0 g/dL and RBC 2.12 106/mm^3^. Consideration was given to initiate emergent TPE; however, this was deferred due to a lack of altered mental status, fever, or severe renal impairment, making TTP somewhat less likely. Serum vitamin B12 levels were undetectable, and daily intramuscular vitamin B12 injections were initiated for seven days followed by weekly injections for four weeks and monthly injections after that. Anti-intrinsic factor antibody serologic testing returned positive confirming the underlying diagnosis of pernicious anemia. After seven days of inpatient vitamin B12 replacement, the patient’s blood count improved significantly and she was safely discharged home. At the time of discharge, her serum hemoglobin was 9.2 g/dL, RBC was 2.63x106/mm^3^, MCV was 106.5 fL, WBC count was 10.5 3.9x103/mm3, and her platelet count improved to 148x103/mm3. The remainder of lab derangements present on admission had also resolved or significantly improved at the time of discharge, including her acute kidney injury, transaminitis, LDH, and hyperbilirubinemia. She was instructed to follow up outpatient for a lifelong continuation of monthly vitamin B12 injections.

**Table 1:** Patient's Lab Work at Presentation LDH = Lactate Dehydrogenase; AST = Aspartate Aminotransferase; ALT = Alanine Transaminase; TSH = Thyroid-Stimulating Hormone; COVID = Coronavirus Disease; PCR = Polymerase Chain Reaction

Lab Work	Patient’s Value	Reference Range
White blood cells	3.9x10^3^/mm^3^	4.0-10.5x10^3^/mm^3^
Platelet count	25x10^3^/mm^3^	150-450x10^3^/mm^3^
Hemoglobin	5.8 g/dL	11.2-15.7g/dL
Red blood cells	1.39x10^6^/mm^3^	3.93-5.22x10^3^/mm^3^
Mean corpuscular volume	119 fL	79.4-94.8fL
Haptoglobin	<20 mg/dL	30-200 mg/dL
LDH	8420 U/L	87-241 U/L
Total bilirubin	4.1 mg/dL	0.2-1.0 mg/dL
Reticulocyte %	7.9%	0.5-1.5%
Reticulocyte index	1.33	≥2
Glomerular filtration rate	46	>59
Corrected serum calcium	8.0 mg/dL	8.5-10.1 mg/dL
AST	229 units/L	15-37 units/L
ALT	68 units/L	12-56 units/L
Serum uric acid	9.8 mg/dL	2.6-6.0 mg/dL
TSH	2.69 uIU/mL	0.36-3.74 uIU/mL
Ferritin	78.8 ng/mL	8-388 ng/mL
Folate	>15 ng/mL	>2.76 ng/mL
COVID PCR	Negative	Negative
Viral hepatitis panel	Negative	Negative
Stool occult blood	Positive	Negative

**Table 2:** PLASMIC Score of the Patient MCV = Mean Corpuscular Volume; INR = International Normalized Ratio; PLASMIC = Platelet count; combined hemoLysis variable; absence of Active cancer; absence of Stem-cell or solid-organ transplant; MCV; INR; Creatinine

PLASMIC Score Calculator		
Platelet count <30 x10^9^L	Yes (+1)	No (0)
Hemolysis - Reticulocyte count >2.5% OR - Haptoglobin undetectable OR - Indirect bilirubin >2.0 mg/dL	Yes (+1)	No (0)
Active cancer (treated for cancer within the past year)	Yes (0)	No (1+)
History of solid-organ or stem-cell transplant	Yes (0)	No (1+)
MCV <90 fL	Yes (0)	No (0)
INR <1.5	Yes (+1)	No (0)
Creatinine <2.0 mg/dL	Yes (+1)	No (0)
Total:	6 points (72%)	

## Discussion

Clinically diagnosing TTP carries a significant burden. Upon making the decision to treat based on suspicion, the diagnostician must elect to place the patient at risk of the first-line treatment. As discussed earlier, the first-line treatment of TTP is placing a dialysis catheter and initiating TPE. Adverse reactions to these treatments include infections and thrombosis as a result of the dialysis catheter itself as well as anaphylaxis from the treatment itself. Another complication of initiating this treatment is the frequent requirement for transportation to alternate facilities as TPE is not readily available in every hospital.

Tools such as clinical assessment and the PLASMIC score are not infallible, which can place patients at risk of being falsely diagnosed with TTP. The PLASMIC score is not only prone to errors, but its accuracy also varies with age (Table 3). In this case, therapy was able to be deferred as the patient was hemodynamically stable, with the concurrent presence of leukopenia which is unexpected in TTP, lack of severe renal failure, and lack of obvious triggers such as starting new medications or history of a recent illness. Initiating a broad workup into various causes of anemia is essential for making a timely diagnosis and avoiding further delay in care.

**Table 3:** PLASMIC Score Accuracy by Age Range PLASMIC = Platelet count; combined hemoLysis variable; absence of Active cancer; absence of Stem-cell or solid-organ transplant; MCV; INR; Creatinine; MCV = Mean Corpuscular Volume; INR = International Normalized Ratio

PLASMIC Score		Age Group (Years)		
		18-39	40-59	≥ 60
≥ 5	Sensitivity	91.4	78.3	76.9
	Specificity	60.0	33.3	50.0
≥ 6	Sensitivity	82.9	65.2	61.5
	Specificity	80.0	75.0	85.7

A literature review elicited a similar case where the patient was diagnosed with pernicious anemia as well [11]. He was found to have significant anemia as well as thrombocytopenia on presentation, which prompted the discussion of TTP as a differential. That patient, however, did not have leukopenia and the thrombocytopenia was not as significant. The unique aspects of this case include the presence of transaminitis as well as renal impairment, which were not present in the published case report. The most notable difference between the two was that the evidence of hemolysis in the other case was less severe. While the LDH was significantly elevated (4050 U/L), the reticulocyte count was 2.48% and the total bilirubin was 1.7. The serum haptoglobin level was undetectable. The calculated PLASMIC score of that patient with the information provided was 4 points, which equates to a 0% risk of TTP. In that case, the PLASMIC score helped avoid the incorrect diagnosis and potentially saved the life of the patient. In our case, however, the PLASMIC score was not enough to rule out the incorrect diagnosis, and we were required to use our broader education and clinical reasoning skills to reach the correct diagnosis.

Two additional cases were discovered in our review of prior case reports in which the patient did receive TPE. In one of the cases, the patient was found to have hemolytic anemia along with altered mentation resulting in a presumptive diagnosis of TTP [12]. It was later discovered, after initiating TPE, that he had nutritional deficiencies in both vitamin B12 and B1. The patient with a PLASMIC score of 5 (Intermediate risk) received one 1,000 mg dose of vitamin B12, solumedrol, and was started on TPE. After four days of TPE, the ADAMTS13 result was found to be negative. He had not benefited from TPE during this time and had not received any thiamine or additional doses of vitamin B12. The second case was of a patient who presented with fatigue, weakness, confusion, dyspnea on exertion, and bilateral lower extremity numbness for two months [13]. She was also found to have hemolytic anemia and was started on TPE with a presumptive diagnosis of TTP. Her PLASMIC score was 3 (Low risk) at presentation. The patient received three days of prednisone and TPE before her vitamin B12 results came back undetectable. Intrinsic factor and TSH were also elevated and the T4 was low. She was given a vitamin B12 replacement and started on Synthroid as well. In these two cases, the patients both received TPE despite not requiring it and did not receive the treatment for the correct diagnoses until a few days had passed. Both cases presented with lower PLASMIC scores than our patient, however, given they presented with altered mentation, the suspicion for TTP was appropriately higher. The TPE may have been warranted in this case for this reason, however, the patients could have also received vitamin B12 injections in addition, given that it is safe to administer while waiting for the lab results to be completed.

## Conclusions

Although this patient had multiple signs and symptoms of TTP, as well as an elevated PLASMIC score, clinical judgment was utilized by multiple subspecialties, making TTP a less likely etiology and thus allowing her to avoid TPE as well as its associated complications. In a patient with no discernible risk factors, it is important to consider a wide variety of etiologies to avoid missing the correct diagnosis. As a result, this patient avoided potentially harmful therapy and underwent the correct and much safer treatment with B12 injections. This case report is intended to demonstrate the need for establishing a broad differential and using a multidisciplinary approach in treating and diagnosing patients with unexpected presentations.
